# Pretransplant Infusion of Donor B Cells Enhances Donor-Specific Skin Allograft Survival

**DOI:** 10.1371/journal.pone.0077761

**Published:** 2013-10-28

**Authors:** Julia Gao, Megan S. Ford. McIntyre, Cheryl A. D'Souza, Li Zhang

**Affiliations:** 1 University of Toronto Transplantation Institute, Toronto General Research Institute, University Health Network, Toronto, Ontario, Canada; 2 Department of Immunology, University of Toronto, Toronto, Ontario, Canada; 3 Department of Laboratory Medicine and Pathobiology, University of Toronto, Toronto, Ontario, Canada; Penn State University, United States of America

## Abstract

Pretransplant donor lymphocyte infusion (DLI) has been shown to enhance donor-specific allograft survival in rodents, primates and humans. However, the cell subset that is critical for the DLI effect and the mechanisms involved remain elusive. In this study, we monitored donor cell subsets after DLI in a murine MHC class I L^d^-mismatched skin transplantation model. We found that donor B cells, but not DCs, are the major surviving donor APCs in recipients following DLI. Infusing donor B, but not non-B, cells resulted in significantly enhanced donor-specific skin allograft survival. Furthermore, mice that had received donor B cells showed higher expression of Ly6A and CD62L on antigen-specific TCRαβ^+^CD3^+^CD4^−^CD8^−^NK1.1^−^ double negative (DN) regulatory T cells (Tregs). B cells presented alloantigen to DN Tregs and primed their proliferation in an antigen-specific fashion. Importantly, DN Tregs, activated by donor B cells, showed increased cytotoxicity toward anti-donor CD8^+^ T cells. These data demonstrate that donor B cells can enhance skin allograft survival, at least partially, by increasing recipient DN Treg-mediated killing of anti-donor CD8^+^ T cells. These findings provide novel insights into the mechanisms underlying DLI-induced transplant tolerance and suggest that DN Tregs have great potential as an antigen-specific immune therapy to enhance allograft survival.

## Introduction

Pretransplant donor specific transfusion or donor lymphocyte infusion (DLI) has been used either alone or in combination with other treatments to prolong graft survival in various animal models and in clinical settings [1]–[6]. However, the mechanism by which DLI induces donor-specific transplantation tolerance is poorly defined. DLI-induced graft survival has been shown to be directly correlated with the infused lymphocytes in the recipients [7]. Nevertheless, which subsets of donor cells are critical for tolerance induction remains controversial [7]–[10].

B cells have long been considered as positive regulators in immune responses contributing to pathogenesis in a variety of immune disorders because of their ability to generate antibodies. However, evidence that B lymphocytes are able to regulate immune responses is accumulating. Convincing data has demonstrated that B cells can be tolerogenic rather than immunogenic in several immune-related diseases [11], [12]. As B cells have been shown to play critical roles in both graft rejection and tolerance, further understanding the role of B cells in transplantation will facilitate the development of novel B cell directed strategies as well as modify previous B cell therapies to achieve donor-specific transplant tolerance [13], [14].

As a subset of regulatory T cells (Tregs), TCRαβ^+^CD3^+^CD4^−^CD8^−^NK1.1^−^ double negative regulatory T cells (DN Tregs) comprise 1–3% of peripheral T lymphocytes in mice and humans [15], [16]. Accumulating evidence has demonstrated that DN Tregs can function as critical immunoregulators in various diseases [17], [18]. It has been shown that DN Tregs can inhibit type 1 diabetes [19], [20], suppress antigen-specific allo- /xeno-reactive syngeneic T cells and induce long-term skin, cardiac and islet graft survival [21]–[23]. Previous studies have demonstrated that DLI activates recipient DN Tregs which are important for suppressing anti-donor T cells and maintaining long-term donor-specific transplantation tolerance [23], [24]. However, the subset of donor cells that is critical for activating DN Tregs and the underlying mechanisms remain obscure.

In this study, we monitored infused donor cells and found that donor B cells, but not DCs, are the major surviving donor APCs in recipients following DLI. Interestingly, infusing purified donor B cells resulted in significantly enhanced donor-specific skin allograft survival. Donor B cells were able to present alloantigen to DN Tregs, induce their proliferation and enhance DN Treg-mediated elimination of anti-donor CD8^+^ T cells. These findings provide novel insights into the role of donor B cells in DLI-induced donor-specific transplant tolerance, and open a new window for using B cells to enhance DN Treg function and allograft survival.

## Materials and Methods

### Ethics Statement

Animals were housed in the Toronto Medical Discovery Tower under specific pathogen-free conditions. The animal use protocol was approved by the University Health Network Animal Care Committee. Animal care was conducted in accordance with the policies and guidelines of the Canadian Council on Animal Care and the Province of Ontario's Animals for Research Act.

### Mice

2C (H-2^b^, expressing the 1B2^+^ anti-L^d^ transgenic TCR) breeders on C57BL/6 (B6) background were kindly provided by Dr. D.H. Loh (Nippon Research Centre, Japan). Dm2 mice, a BALB/c L^d^-loss mutant, (H-2D^d^, K^d^, L^0^) were bred with 2C mice to create 2C_F1_ mice (anti-L^d^ TCR, H-2^b/d^, L^d−^) or with B6 mice to create (B6×dm2)_F1_ (H-2^b/d^, L^d−^) mice. B6, GFP^+^B6, BALB/c and SJL (H-2^s^) mice were purchased from the Jackson Laboratory. CBy and GFP^+^CBy (H-2^b/d^, L^d+^) mice were created by crossing B6 or GFP^+^B6 (H-2^b^) to BALB/c (H-2^d^) mice. All mice were housed in specific pathogen-free conditions at the University Health Network (Toronto, ON). All experiments were approved by the University Health Network animal care committee.

### Antibodies and reagents

Anti-L^d^ monoclonal antibody (mAb) and 1B2 mAb that recognizes the 2C_F1_-TCR were produced and purified in our lab as described previously [16]. Other mAbs include anti-CD3, anti-CD4, anti-CD8, anti-CD19, anti-CD62L, anti-DX5, anti-CD25, anti-CD44, anti-Ly6A, and the reagents used to identify dead cells including Annexin V and PI (propidium iodide) were purchased from eBioscience (San Diego, CA). Anti-NK1.1, anti-CD11c and anti-CD11b mAbs were purchased from BD PharMingen (BD Biosciences, Canada).

### DLI and skin grafting

The 2C_F1_ recipient mice (L^d−^) were left untreated or i.v. infused with 30×10^6^ of a mixture of spleen and LN cells, purified B or non-B cells collected from naïve CBy or GFP^+^CBy mice (L^d+^). Skin grafting was preformed as previously reported [16]. Briefly, 7 days following DLI, each recipient mouse received a sex-matched skin graft from either CBy (donor-specific) or SJL (third party) mice, and a 2C_F1_ skin graft (syngeneic). A piece of donor tail skin about 1×0.5 cm^2^ with a thickness including the epidermis and most of the dermis was removed with a sharp scalpel and transferred to the sides of the recipient's tail, from which an equivalent amount of skin had been removed. The grafts were covered with a clear spray bandage and further protected with a light, loosely fitted transparent glass tube. Grafts were monitored by visual inspection daily for the first 2 weeks and twice a week thereafter until rejection. A graft was considered rejected when more than 90% was necrotic. Mice received the same skin grafts without DLI were as controls.

### B and non-B cell purification

Cells from spleen and LN of CBy mice were collected and red blood cells were lysed. Cells were incubated at 4°C with anti-CD19 (FITC) mAb for 10 minutes, washed and incubated with anti-FITC MACS beads for 15 minutes, then washed and passed through the AutoMACS to positively select the CD19^+^ or negatively select CD19^−^ population following the manufacturer's directions (Miltenyi Biotech, Auburn, CA). The B Cell Isolation Kit (Miltenyi Biotech, Auburn, CA) was also used to isolate untouched resting B cells according to manufacturer's instruction. The purity and viability of CD19^+^, CD19^−^ cells and resting B cells used in the experiments were more than 95%.

### DN Treg and CD8^+^ T cell isolation

To purify DN Tregs, LN and spleen cells from 2C_F1_ mice were collected and red blood cells were lysed. To enrich the DN Tregs, CD4^+^ and CD8^+^ T cells were depleted by incubating the cells with anti-CD4 (RL-172-4, rat IgM) and anti-CD8 (3.168, rat IgM) depleting mAbs on ice for 30 minutes, followed by incubation with Low-Tox Rabbit Complement (Cedarlane Laboratories Limited, Hornby, ON) at 37°C for 45 minutes. Remaining cells were cultured with irradiated CBy splenocytes in complete medium (CM), which is α-MEM medium supplemented with 10% FCS, Penicillin (100 U/ml, Sigma), Streptomycin (100 µg/ml, Sigma), L-glutamine (2 mM, Sigma), 2-mercaptoethanol (50 µM, Sigma), 50 U/ml rIL-2 and 30 U/ml rIL-4. After 4 days culture, cells were stained with anti-CD4, anti-CD8 and anti-NK1.1 mAbs on ice for 30 minutes followed by incubation with MACS beads (Miltenyi Biotech, Auburn, CA) at 4°C for 15 minutes, and then washed and passed through an autoMACS column (Miltenyi Biotech, Auburn, CA) to deplete CD4^+^CD8^+^NK1.1^+^ cells. The remaining CD4^-^CD8^-^NK1.1^-^1B2^+^ cells had greater than 90% purity and viability.

To purify the CD8^+^ T cell population, cells from spleen and LN of 2C_F1_ mice were collected and red blood cells were lysed as described before. Cells were incubated at 4°C with anti-CD8 (FITC) mAb for 10 minutes, washed and incubated with anti-FITC MACS beads for 15 minutes, then washed and passed through the AutoMACS to positively select the CD8^+^ population following the manufacturer's directions (Miltenyi Biotech, Auburn, CA). The purity and viability of CD8^+^ T cells used in the experiments were more than 95%.

### APC cell culture

LPS activated-B cells were obtained by culturing CBy-L^d+^ splenocytes in the presence of 10 µg/ml LPS (E. coli, Sigma-Aldrich, Oakville, ON) for 24 hours. Cultures were found to be greater than 90% CD19^+^. BM derived mature DCs (mDCs) were obtained as previously described [25]. On day 8–9, non-adherent cells were collected and used as iDCs. To induce DC maturation, 0.1 µg/ml LPS was added into the culture. The non-adherent cells were harvested the next day and were used as mDCs. Cell surface marker expression was analyzed by flow cytometry (Cytomics™ FC 500, Beckman Coulter). Over 90% of the DCs used in experiments were CD11c^+^ cells. Viability of DCs was >95%, as determined by staining with PI.

### Proliferation Assay

Primary DN Tregs were stimulated *in vitro* by purified B cells in the presence of either 10 µM QL9 (QLSPFPFDL) or P1A (LPYLGWLVF) peptides. Cell proliferation was determined by pulsing with 5 µCi/ml ^3^H-TdR for 18 hrs on day 3 of culture. Cells were harvested onto 96 well filter plates and ^3^H-TdR labelling was counted on a TOPCOUNT plate reader (Perkin Elmer Life and Analytical Science, Woodbridge, ON).

### Cytotoxicity Assay

CD8^+^ T cells purified from naïve (B6×dm2)_F1_ mice were stimulated by either irradiated CBy (L^d+^, donor-specific) or SJL (H-2^S+^, third party) splenocytes for 4 days and used as targets. DN Tregs purified from spleen and LNs of 2C_F1_ mice 4 days after receiving purified B or non-B lymphocyte infusion, were stimulated by irradiated L^d+^ B or non-B cells *ex vivo* for 4 days, purified and co-incubated with CD8^+^ T cell targets at varying ratios for 24 hours. Cells were then stained with anti-CD8 mAb followed by Annexin V and PI. CD8^+^ cells were gated and the percentages of Annexin V^+^ and/or PI^+^ cells were determined by flow cytometry.

### Data Analysis

Flow cytometry data were analyzed and presented with FlowJo 7.6 (Tree Star). Statistical analysis and graphical presentation of data were performed with Prism 5.0 (GraphPad, La Jolla CA). The means of two groups were compared with Student's *t* test; Two-way ANOVA was used to analyze the variance between two or more groups with respect to two or more factors; Graft survival was determined using the log-rank test. *p*<0.05 was considered statistically significant.

## Results

### Pretransplant infusion of donor cells induces long-term single MHC locus-mismatched donor-specific skin allograft survival

**Figure 1:**
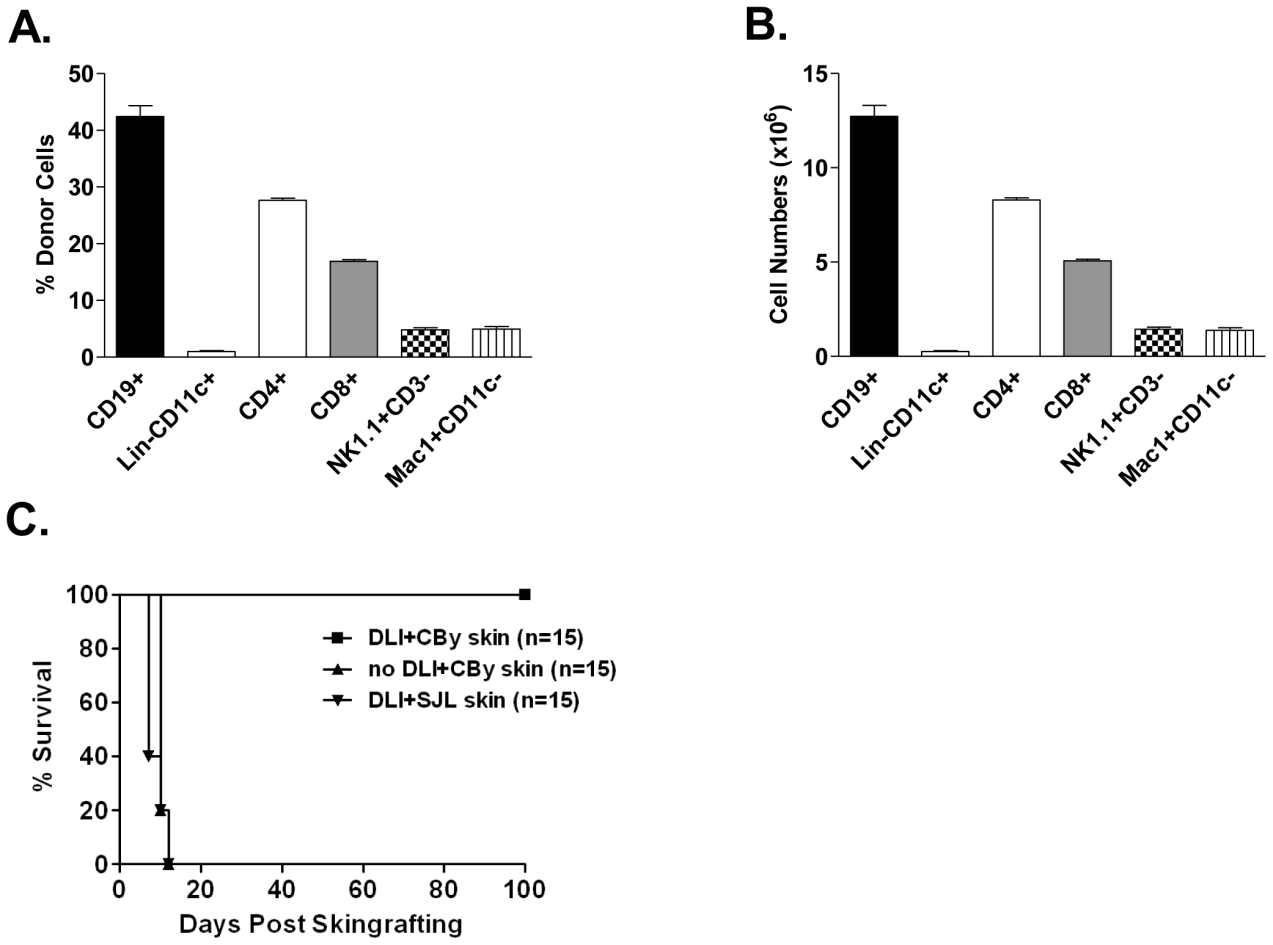
Pretransplant infusion of donor cells induces long-term single MHC locus-mismatched donor-specific skin allograft survival. (**A–B**) Cells from the LNs and spleen of naïve GFP^+^CBy mice were stained with mAbs against CD4, CD8, CD11c, Mac1, Lin (lineage markers, including CD3, CD19 and DX5) and NK1.1. Percent (**A**) and total number (**B**) of different subsets of donor cells were assessed by flow cytometry. (**C**) 2C_F1_ (anti-L^d^-2C-TCR^+^, H-2^b/d^, L^d−^) recipient mice were i.v. injected with 3×10^7^ spleen and LN cells (▪, ▾) from GFP^+^CBy (H-2^b/d^, L^d+^) donors. Seven days later, recipient mice were transplanted with sex-matched CBy (L^d+^, ▪, n = 15) or SJL (H-2^s+^, ▾, n = 15) skin grafts. 2C_F1_ mice that were transplanted with CBy skin grafts without pretransplant DLI were used as control (▴, no DLI, n = 15). Graft survival was monitored by visual inspection daily for the first 2 weeks and twice a week thereafter. Representative data are shown from at least three independent experiments.

Pretransplant infusion of donor spleen and lymph node (LN) cells, either alone or together with other treatments such as anti-CD4 monoclonal antibody (mAb), has been shown to benefit donor-specific allo- and xeno-graft survival [23], [24]. To identify the beneficial donor cell subsets, we used pan-GFP^+^ mice as lymphocyte donors to track donor cells in GFP^-^ recipients. GFP^-^ 2C_F1_ mice (H-2^b/d^, L^d−^, which express a transgenic TCR that specifically recognizes L^d^) were infused with GFP^+^CBy (H-2^b/d^, L^d+^) donor lymphocytes. Before infusion, donor cell composition was examined and is shown in Fig. 1A-B. As expected, CD19^+^ B cells were the major subset of donor cells, which was followed by CD4^+^ and CD8^+^ T cells. DCs, NK cells and macrophages were also present but comprised much smaller proportions. One week after infusion, all mice were transplanted with skin allografts from both the lymphocyte donor strain, CBy, and a third-party strain, SJL. Graft survival was monitored. As shown in Fig. 1C, mice that did not receive pretransplant DLI rejected CBy skin grafts within 2 weeks. However, all mice which had received CBy cells accepted skin allografts from CBy mice permanently (>100 days) and rejected skin grafts from SJL mice within 2 weeks. These data demonstrate that pretransplant DLI can induce long-term donor-specific skin allograft survival in this model.

### Tracking donor cells in recipient mice

**Figure 2:**
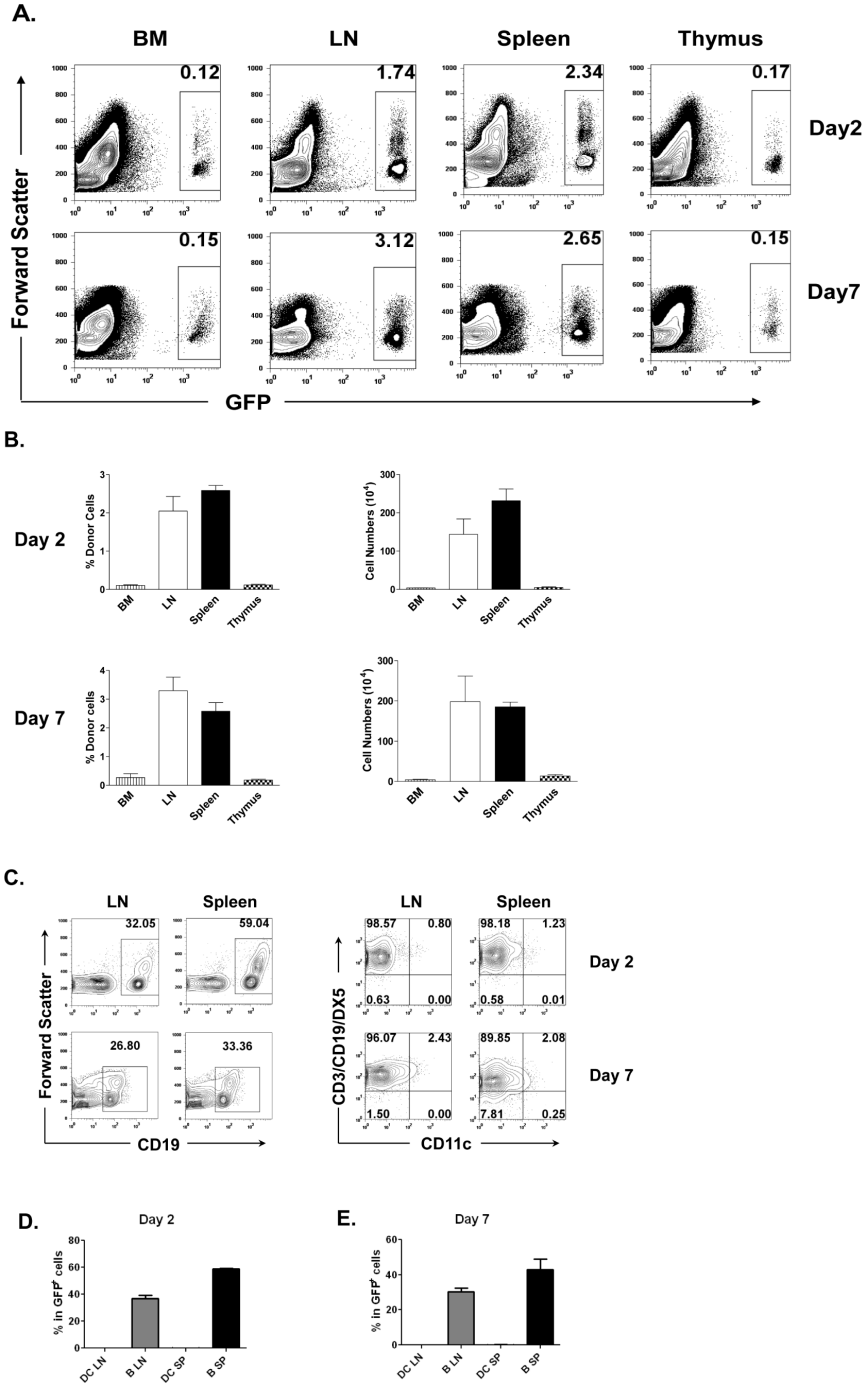
Tracking donor cells in recipient mice. 2C_F1_ mice were infused with 6×10^7^ allogeneic GFP^+^CBy (L^d+^) cells. Two or seven days later, cells were harvested from recipient thymus, bone marrow (BM), spleen and LNs. (**A**) Numbers in the graphs indicate % GFP^+^ donor cells in each organ. (**B**) Bar graphs show mean ± SEM of the percentages (left panels) or total numbers (right panels) of donor cells at 2 and 7 days following DLI from 3 independent experiments. (**C–E**) Cells were stained with mAbs against CD19, CD11c, CD3 and DX5, and analyzed by flow cytometry. (**C**) The Representative plots were gated on GFP^+^ cells and showed % of each subset of cells in donor cell population on day 2 and 7 following DLI. (**D**) and (**E**) show mean ± SEM percentages of donor B cells and DCs in recipient LN and spleen on day 2 (**D**) or day 7 (**E**) following DLI. These experiments were repeated at least three times and representative data are shown.

In order to understand the mechanisms involved in DLI-induced donor-specific skin allograft survival, we tracked the donor cells in recipient mice. As shown in Figs. 2A and 2B, surviving donor cells were mostly found in the LNs and spleen, but were few in the bone marrow and thymus. This suggests that the donor cells interacting with recipient cells in peripheral lymphoid organs may contribute to the observed long-term acceptance of donor skin allografts. To determine the survival and importance of donor cell subsets in DLI-induced transplantation tolerance, subsets of GFP^+^ donor cells in the recipient bone marrow, thymus, spleen and LNs were studied on day 2 and 7 after DLI. As shown in Fig. 2C-E, few donor DCs were detected in either recipient spleen or LNs, however, there was a large population of CD19^+^ cells remaining at these time points in both LNs and spleen. These data suggest that donor B cells may play an important role in the tolerance induction as observed in Fig 1C.

### Donor B cells are more effective at enhancing donor-specific skin allograft survival than non-B cells

**Figure 3:**
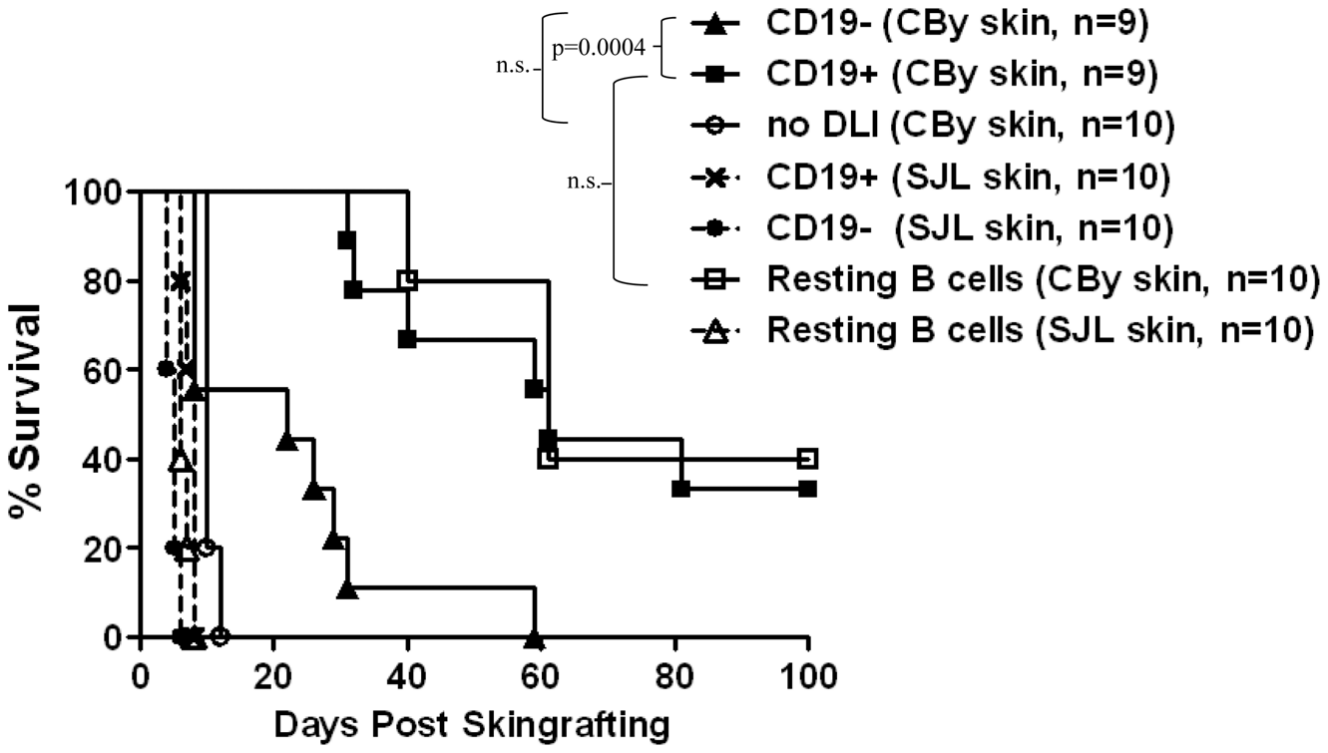
Pretransplant infusion of donor B cells significantly enhances donor-specific skin allograft survival. 2C_F1_ mice were left untreated (no DLI, ○), or i.v. infused with either 3×10^7^ CD19^+^ (▪,×) or CD19^−^ (▴, •) cells, or untouched naïve B cells (CD43^-^CD4^-^Ter119^−^ cells, Δ, □) from CBy mice. Seven days later, recipients were transplanted with either sex-matched L^d+^ CBy (▪, n = 9; ▴, n = 9; □, n = 10; ○, n = 10), or SJL skin allografts (×, n = 10; •, n = 10; Δ, n = 10). Graft survival was monitored for more than 100 days. The mean survival time was calculated for each treatment and the log-rank test was used to analyze significance.

Accumulating evidence suggests that B cells can promote immune tolerance in various disease models [12], albeit their role in transplantation tolerance has not been well defined. To determine whether donor B cells are important for enhancing donor-specific skin allograft survival, lymphocytes from naïve CBy (L^d+^) mice were sorted into CD19^+^ and CD19^−^ populations and infused into 2C_F1_ (L^d−^) mice. One week later, all mice were transplanted with a syngeneic skin graft (negative control) together with a skin allograft from either lymphocyte donor CBy mice or 3^rd^ party SJL mice. As shown in Fig. 3, mice that did not receive DLI rejected CBy skin allografts within 12 days with a Median Survival Time (MST) of 10 days. All 3^rd^-party SJL skin grafts were rejected within 8 days regardless of the type of donor cells they received prior to transplantation. Infusing CD19^−^ CBy lymphocytes slightly prolonged the survival of donor specific skin grafts compared to the no DLI group, but the difference was not statistically significant (*p* = 0.7301). Interestingly, 2C_F1_ mice that received CBy CD19^+^ B cells showed significant enhancement of donor specific skin graft survival (MST = 61 days) compared to those that received CBy CD19^−^ cells (MST = 22 days, *p* = 0.0004). Three out of nine mice accepted donor specific skin allografts for over 100 days (Fig.3). These data indicate that infusion of CD19^+^, but not CD19^−^ donor cells, can enhance donor-specific, but not third party, skin allograft survival.

In the above experiment, we used anti-CD19 mAb coated magnetic beads to positively select CD19^+^ cells for i.v. injection. To exclude the potential effect of this selection procedure on skin graft survival, untouched naïve B cells were purified by depleting CD43^+^CD4^+^Ter119^+^ cells and i.v. injected into 2C_F1_ mice. The recipients were then transplanted with either a CBy or an SJL skin allograft together with a syngeneic skin graft. As shown in Fig. 3, mice receiving untouched naïve B cells showed similar CBy skin graft survival as mice receiving B cells purified by positive selection of CD19^+^ cells. 4 out of 10 mice which received negatively selected B cells had their CBy skin grafts survive for 100 days. Taken together, these data demonstrate that pretransplant infusion of donor B lymphocytes is significantly more effective at enhancing donor-specific skin allograft survival than infusion of non-B donor lymphocytes. However, B cells alone were not able to induce complete tolerance as observed in Fig. 1C.

### DN Tregs can be activated by allogeneic donor B cells in an antigen-specific manner and preferentially acquire alloantigen from B cells

**Figure 4:**
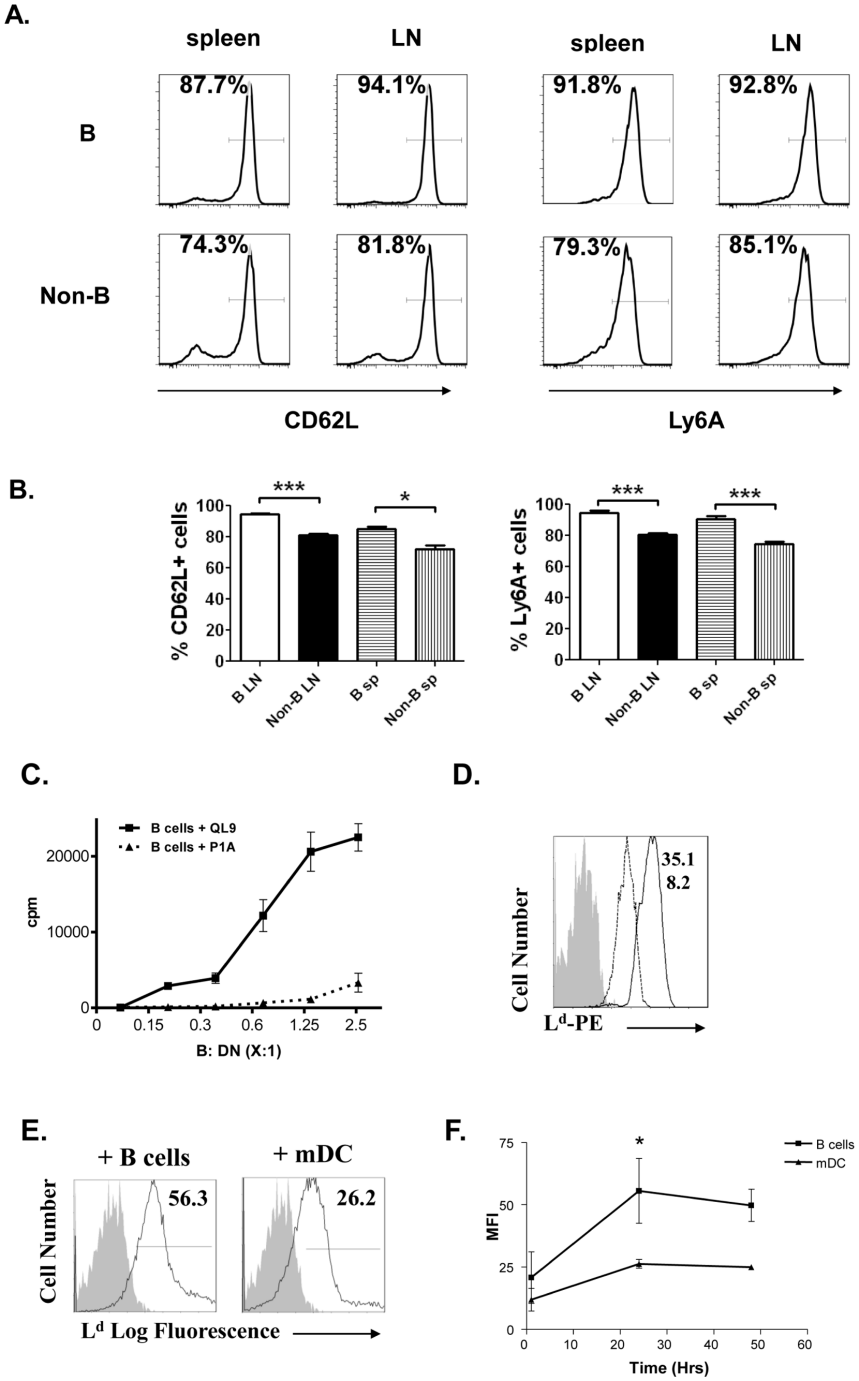
DN Tregs can be activated by allogeneic donor B cells in an antigen-specific manner and preferentially acquire alloantigen from B cells. **(A–B)** 2C_F1_ mice were infused with 3×10^7^ purified B or non-B cells from CBy mice as described in Fig. 3. Cells were harvested from LNs and spleens of the recipients 7 days later, stained with mAbs against CD4, CD8, NK1.1 and 1B2 in combination with activation markers CD62L or Ly6A. **(A)** Representative histograms from one of three independent experiments show percentages of CD62L^+^ and Ly6A^+^ populations on CD4^-^CD8^-^NK1.1^-^1B2^+^ antigen-specific DN Tregs. **(B)** Bar graphs summarized the percentages of CD62L^+^ and Ly6A^+^ cells from 3 independent experiments. **(C)** 2C_F1_ DN Tregs were co-cultured with increasing doses of irradiated CBy (L^d+^) B cells that had previously been activated with 10 µg/ml LPS for 24 hours in the presence of either 2C_F1_-TCR non-specific P1A (dashed line) or specific QL9 (solid line) peptides that bind to MHC class I – L^d^. Proliferation of DN Tregs was assessed by ^3^H-TdR labelling for 18hrs after 3-days of coculture. **(D)** LPS activated B cells (solid line) and mDCs (dashed line) were stained with mAb specific for L^d^. Isotype control was used as a negative control (shaded histogram). The numbers show the MFI of L^d^ expression on B cells (top) and mDCs (bottom). **(E–F)** 2C_F1_ DN Tregs were co-incubated with L^d+^ B cells (CD19^+^) or mDCs (CD11c^+^, MHCII^high^, CD86^high^). **(E)** Representative histograms show L^d^ expression on the surface of DN Tregs after 48 hrs of co-incubation with either B cells (left panel) or mDCs (right panel). Isotype control was used as a negative control (shaded histogram). The numbers are MFI of the L^d^ alloantigen expressed on the DN Tregs. **(F)** MFI of L^d^ on the surface of 1B2^+^DN Tregs at 1, 24, and 48 hours after coculture with each cell type. These experiments were repeated at least 3 times and the results were analyzed by Two-way ANOVA. 0.01<*p<0.05, ***p<0.0001

It has been shown previously that DN Tregs play a key role in DLI-induced donor-specific transplantation tolerance [23], [24]. In order to understand the mechanisms by which infusion of donor B cells prolonged donor-specific allograft survival, we first examined whether donor B cells can function as APCs to activate recipient DN Tregs. Although both B and non-B cell infusion resulted in similar increased expression of CD25 and CD44 on recipient DN Tregs (data not shown), CD62L^+^ and Ly6A^+^ expression on recipient DN Tregs were significantly increased in mice that received donor B cells compared to those that received non-B cells (Fig. 4A-B). We next determined whether donor B cells could directly prime DN Tregs in an antigen-specific manner. As shown in Fig. 4C, 2C_F1_ DN Tregs proliferated significantly in response to B cells which had been pulsed with 2C TCR specific peptide QL9. However, no proliferation of DN Tregs was observed when they were stimulated with B cells pulsed with TCR non-specific P1A peptides (Fig. 4C, *p*<0.0001). This data demonstrates that B cells can present alloantigen and prime DN Treg proliferation in an antigen-specific fashion.

Previous studies have shown that acquisition of alloantigens is required for both human and mouse DN Treg-mediated antigen-specific immune suppression [15], [16]. We found that activated donor B cells expressed higher levels of alloantigen L^d^ on their surface than mature DCs (mDCs) (Fig. 4D). To determine whether higher L^d^ expression would facilitate DN Treg acquisition of alloantigen from B cells, we compared acquisition levels achieved by 1B2^+^DN Tregs in the presence of either activated B cells or mDCs from allogeneic L^d+^ mice. The DN Tregs that were cocultured with B cells showed significantly increased expression of L^d^ on their surface when compared to those cocultured with mDCs (Fig. 4E–F). These data suggest that although DN Tregs can acquire alloantigens from both mDCs and activated B cells, interaction with the latter resulted in higher acquisition and expression of L^d^ by DN Tregs.

### Recipient DN Tregs activated by donor B cells show higher toxicity towards anti-donor CD8^+^ T cells in an antigen-specific fashion

**Figure 5:**
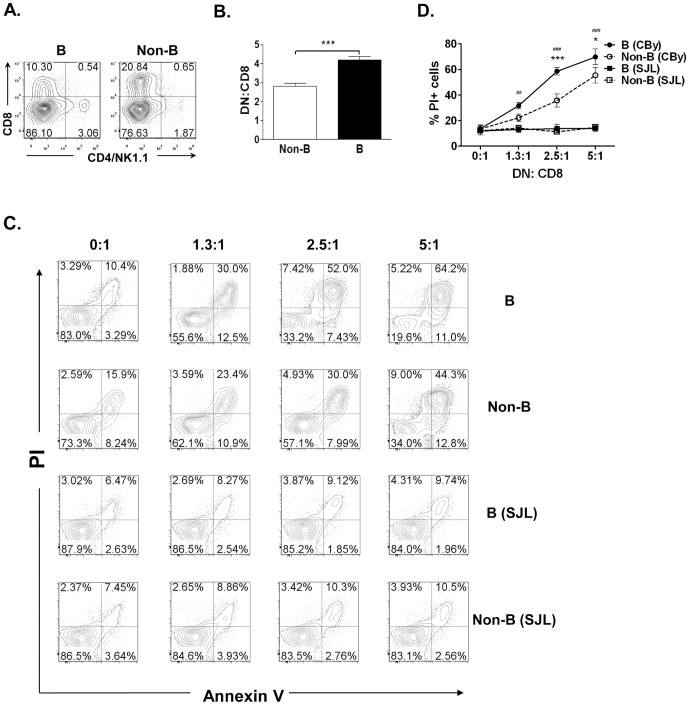
Recipient DN Tregs activated by donor B cells show higher toxicity towards anti-donor CD8^+^ T cells in an antigen-specific fashion. (**A, B**) 2C_F1_ mice were i.v. injected with 3×10^7^ B or non-B cells isolated from CBy mice. Cells were harvested from spleen and LNs on day 7, stained with anti-CD4, anti-CD8, anti-NK1.1 and anti-1B2 mAbs, then examined by flow cytometry. (**A**) Donor-specific DN Tregs and CD8^+^ T cells were gated on 1B2^+^ population. (**B**) Based on the flow cytometry data, 1B2^+^DN Treg to1B2^+^ CD8 ratio from a total of 8 mice from 3 independent experiments was calculated. (**C**) DN Tregs were isolated from 2C_F1_ mice 7 days after infusion of either CBy B or non-B cells followed by restimulation *ex vivo* of either CBy or SJL B or non-B cells before being used as effectors. Activated anti-L^d+^ or anti-H-2^s^ CD8^+^ T cells were used as targets. Effectors and targets were cultured either alone or together at varying ratios as indicated. After 24 hrs, cultures were stained with anti-1B2, anti-CD8 mAbs, Annexin V and PI. Percentages of Annexin V^+^ and PI^+^ cells in 1B2^+^CD8^+^ gated cells are shown in (**C**). Data shown in (**D**) are from 3 independent experiments. Two-way ANOVA with Bonferroni's post-test was used to analyze the data. The asterisk symbol (*) shows significance between B (CBy) and Non-B (CBy) graphs. The hash symbol (#) shows significance between B (CBy) and B (SJL) graphs. *p<0.05, **p<0.01, ***p<0.001

Ly6A has been shown to be required for DN Treg-mediated cytotoxicity toward CD8^+^ T cells [26]. As Ly6A expression on DN Tregs was significantly increased in mice that received donor B cell infusion (Fig. 4A–B), we addressed the question of whether infusion of donor B cells would increase the ability of DN Tregs to kill syngeneic anti-donor CD8^+^ T cells *in vivo*. To this end, 2C_F1_ mice were injected with either naïve B or non-B lymphocytes from CBy mice and sacrificed 7 days later. We found that mice that had received B cell infusion showed a significant decrease in 1B2^+^CD8^+^ T cells, concomitant with an increase in 1B2^+^DN Tregs, leading to significantly increased antigen-specific DN to CD8 T cell ratio (Fig. 5A–B). These findings further suggest that DN Tregs that were activated by donor B cells may possess enhanced cytotoxicity toward anti-donor CD8^+^ T cells. To directly test this hypothesis, DN Tregs isolated from spleen and LNs 4 days after infusion of either donor B or non-B cells were stimulated *ex vivo* by either irradiated L^d+^ B cells or non-B cells before being used as putative effectors. CD8^+^ T cells purified from naïve (B6×dm2)_F1_ (L^d-^) mice were stimulated by irradiated L^d+^ splenocytes and used as targets. As shown in Fig. 5C-D, both non-B cell and B cell activated DN Tregs were able to kill CD8^+^ T cells effectively, however, donor B cell primed DN Tregs showed significantly higher cytotoxicity toward anti-donor CD8^+^ T cells in an antigen-specific manner than those primed with donor non-B cells (*p*<0.001). Furthermore, the DN Tregs activated by either CBy B or non-B cells showed no cytotoxicity to either B or non-B SJL lymphocyte-activated CD8^+^ T cells. Collectively, these findings suggest that infusion of donor B cells may facilitate donor-specific skin allograft survival by activating recipient antigen-specific DN Tregs which then kill anti-donor CD8^+^ T cells in an antigen-specific manner.

## Discussion

Numerous reports have shown that pretransplant exposure to donor antigens through injection of donor leukocytes or blood transfusion either alone or in combination with other treatments can facilitate donor-specific graft survival. To dissect the mechanisms involved in this phenomenon, we used GFP^+^ mice as donors to investigate the spacial and temporal kinetics of infused donor cells in recipients. Consistent with a previous report using GFP^-^ mice [16], pretransplant infusion of GFP^+^ MHC class I L^d^-mismatched lymphocytes induced permanent acceptance of donor-specific skin allografts in all recipients (Fig. 1C). Since all non-DLI-treated mice rejected L^d+^ skin grafts and all the mice that were treated with GFP^+^L^d+^ DLI rejected their 3^rd^-party SJL skin allografts within 2 weeks (Fig. 1C), these results indicate that pretransplant infusion of unfractionated donor lymphocytes can induce donor-specific transplantation tolerance in this MHC class I L^d^-mismatched model.

Several studies have investigated the subsets of donor cells that might be important for inducing donor-specific transplantation tolerance in minor antigen-mismatched models and the results remain controversial. Johnson reported that infusion of donor T cells alone was not sufficient to achieve tolerance to male H-Y antigens [8]. Sheng et al. found that infusion of donor T cells together with Mac-1^+^ cells induced tolerance toward male antigens [10]. On the other hand, Fuchs and Matzinger reported that infusion of resting donor B cells could induce long term survival of male H-Y^+^ skin grafts in female mice [27]. The interaction of FasL, expressed on the infused B cells and Fas expressed on the recipient T cells was essential in DLI-induced tolerance to H-Y antigens [28]. Whether infusion of donor B cells alone is necessary or sufficient to attain MHC-mismatched donor-specific allograft survival was unknown. We found that a single injection of naïve donor B cells, purified by either positive or negative selection, at 7 days prior to transplantation resulted in significantly prolonged donor-specific skin allograft survival (MST = 61 days) compared with infusion of non-B cells (MST = 22 days, p = 0.0004) (Fig. 3). In both treatment groups, 3^rd^ party skin grafts were rejected within 2 weeks. These data provide direct evidence supporting an important role of donor B cells in enhancing donor-specific skin allograft survival in an MHC class I-mismatched model.

Interestingly, while infusing non-fractionated splenocytes induced permanent donor-specific allograft acceptance (Fig. 1C), infusion of the same number of B cells by two different purification methods could only prolong graft survival as the majority of donor skin allografts were eventually rejected (Fig. 3). This was not due to insufficient number of B cells nor donor antigens as even when 60×10^6^/mouse purified B cells were infused, complete tolerance was not achieved (data not shown). Furthermore, infusion of non-B cells was also able to enhance allograft survival compared to no DLI, however, the difference was not statistically significant (Fig. 3). Taken together, these data suggest that although B cells are superior to non-B cells in enhancing donor-specific skin allograft survival, infusion of B cells alone is not sufficient to induce permanent skin allograft acceptance. The type of cells in the non-B cell portion that can facilitate B cell tolerogenic function requires further investigation.

In searching for the mechanisms by which donor B cells may contribute to enhanced donor-specific skin allograft survival, we found that donor B cells, but not DCs, were the major surviving donor APCs following DLI. Large numbers of donor B cells were found in the spleen and LNs of the recipients both on day 2 and day 7 following DLI (Fig. 2C–E). In contrast, almost no donor CD11c^+^CD3^-^CD19^-^DX5^-^ DCs were detected in recipient LNs and spleen (Fig. 2C–E). In addition, recipient DN Tregs showed higher expression of CD62L and Ly6A when recipients were infused with donor B cells compared to when they received non-B cells (Fig. 4A–B). While Ly6A has been shown to be required for DN Treg-mediated cytotoxicity toward CD8^+^ T cells [26], expression of cell adhesion molecule CD62L may be required for their efficient recirculation to facilitate their regulatory function [29]. Furthermore, B cells are able to induce DN Treg proliferation in an antigen-specific fashion (Fig. 4C). These findings suggest that infused donor B cells may facilitate recipient DN Treg activation.

Antigen-activated DN Tregs have been shown to play important roles in inducing donor-specific transplantation tolerance in various models [21], [24], [30]. Furthermore, DN Tregs are able to suppress B cells and NK cells through the perforin/granzyme pathway for tolerance achievement [31]–[33]. Recently DN Tregs were found to express high levels of CTLA4 which could down regulate CD80/CD86 on mDCs [25]. DN Tregs could also kill mDCs and activated B cells through the Fas-FasL or the perforin pathway [25], [31]. So far the immune suppression mediated by mouse, rat and human DN Tregs were found to be antigen-specific [17]. Recent evidence indicates that DN Tregs are able to acquire MHC-peptides from APCs through trogocytosis, and that expression of acquired allo-MHC-peptides on the surface of DN Tregs is critical for their cytolysis of antigen-specific CD8^+^ effector cells [34]. This may be one of the mechanisms by which DN Tregs convey antigen-specific suppression. Here we found that DN Tregs can acquire and express higher levels of donor antigens from B cells than from DCs (Fig. 4E–F). In addition, recipient DN Tregs activated by donor B cells were able to kill donor-specific CD8^+^ T cells more effectively than those activated by non-B cells or by 3^rd^-party B cells (Fig. 5C–D). These data further support the notion that donor B cells may program recipient DN Tregs to enhance their cytotoxicity toward anti-donor CD8^+^ T cells leading to a higher DN Treg to CD8^+^ T cell ratio following DLI (Fig. 5A–B), which could ultimately result in enhancing donor-specific skin allograft survival as observed in this animal model. Consistent with our findings, a recent study also suggested that a higher DN Treg to CD8 ratio in bone marrow transplant patients correlated with a reduced severity of graft versus host disease [35].

Taken together, we demonstrate that pretransplant infusion of donor B cells can prolong skin allograft survival in an antigen-specific fashion, although this treatment is not sufficient to induce complete tolerance. The infused donor B cells may interact with donor-specific DN Tregs, which results in an increased DN Treg-mediated killing of CD8^+^ T cells and contributes to the enhancement of donor-specific skin allograft survival. These findings provide new insights into the mechanisms underlying DLI-induced transplant tolerance and suggest that DN Tregs have great potential as an antigen-specific immune therapy to enhance allograft survival.
